# Enhanced Vaccine Effectiveness during the Delta Phase of the COVID-19 Pandemic in the Medicare Population Supports a Multilayered Prevention Approach

**DOI:** 10.3390/biology11121700

**Published:** 2022-11-24

**Authors:** Bettina Experton, Adrien Elena, Christopher S. Hein, Dale Nordenberg, Peter Walker, Blake Schwendiman, Christopher R. Burrow

**Affiliations:** 1 Humetrix Inc., Del Mar, CA 90214, USA; 2 Thriive, Inc., Bronx, NY 10463, USA; 3 US Navy, Washington, DC 20376, USA

**Keywords:** COVID-19/epidemiology/prevention & control, SARS CoV-2, Delta variant, B.1.617.2, COVID-19/breakthrough infection, BNT162b2 vaccine, mRNA-1273 vaccine, vaccine effectiveness, Medicare, aged 65 and over, multilayered prevention

## Abstract

**Simple Summary:**

Almost three years into the pandemic, older individuals still account for the vast majority of COVID-19 related deaths. While most older adults in the United States have been fully vaccinated, many have not yet completed the recommended course of booster vaccinations. To address U.S. data gaps on Vaccine Effectiveness (VE) in this high-risk population and to best formulate effective booster and other prevention policies, we conducted an observational study of VE in a 17 million Medicare population cohort including 5.7 million fully vaccinated individuals during the Delta variant phase of the pandemic. We observed significant VE in this higher risk population against infections and more so against hospitalizations, apparent waning vaccine-induced immunity, and found added protection from prior COVID-19 infection. We also uniquely report that VE increased over a period of weeks as the Delta surge progressed, suggesting that in addition to the critical protective role of vaccination, individual behavioral factors, such as increased masking or social distancing, likely contributed to this VE increase. Our results emphasize the need for a multipronged prevention strategy to combat the ongoing COVID-19 pandemic including not only vaccination but also complementary preventive measures to reduce the risk of infection, especially for individuals at the highest risk.

**Abstract:**

Throughout the pandemic, individuals 65 years and older have contributed most COVID-19 related deaths. To best formulate effective vaccination and other prevention policies to protect older adults, large scale observational studies of these higher risk individuals are needed. We conducted a Vaccine Effectiveness (VE) study during the B.1.617.2 Delta variant phase of the pandemic in July and August 2021 in a cohort of 17 million Medicare beneficiaries of which 5.7 million were fully vaccinated. We found that individuals fully vaccinated with the Pfizer-BioNTech BNT162b2 and Moderna mRNA-1273 vaccines in January 2021 had 2.5 times higher breakthrough infections and hospitalizations than those fully vaccinated in March 2021, consistent with waning of vaccine-induced immunity. Measuring VE weekly, we found that VE against hospitalization, and even more so against infection, increased from July 2021 through August 2021, suggesting that in addition to the protective role of vaccination, increased masking or social distancing might have contributed to the unexpected increase in VE. Ongoing monitoring of Medicare beneficiaries should be a priority as new variants continue to emerge, and the VE of the new bivalent vaccines remains to be established. This could be accomplished with a large Medicare claims database and the analytics platform used for this study.

## 1. Introduction

Two and half years into the pandemic, the world has suffered over six million COVID-19 related deaths, with more than one million fatalities in the United States alone [1]. In the U.S., the vast majority of severe COVID-19 infections and fatalities have been observed among older adults, with individuals over age 65 representing three quarters of COVID-19 related deaths [2]. The availability of vaccines and new therapeutics has led to reduced morbidity and mortality from severe disease [3] in the overall population and in more vulnerable groups including older adults [4,5,6,7] who in 2022 are contributing to an even higher proportion of COVID-19 related mortality [8]. However, apparent waning of vaccine-induced immunity [9] has led to repeat booster campaigns [10,11,12] for older adults, even if they have the highest vaccination rates of the overall US population, with 94% of the 65 years of age and over having completed their first vaccine series [13].

To best formulate COVID-19 vaccine booster policies and succeed in individual participation in vaccine booster campaigns, the U.S. government has identified the need for large scale critical population health datasets that link an individual’s history of COVID-19 vaccination and COVID-19 testing, as well as their clinical, demographic, and socio-economic data to monitor and analyze COVID-19 infections, related hospitalizations, and deaths. In consequence, the U.S. government has had to heavily rely on Vaccine Effectiveness (hereafter, VE) epidemiologic analyses conducted abroad [10,11,14,15]. The Centers for Disease Control and Prevention (CDC) now recognizes its shortcomings during the COVID-19 pandemic, and proposes to address these problems in its recently announced overall agency reform [16]. This paucity of population health data is due in part to the inability, in most instances, to link at the individual level, vaccination, COVID-19 testing, clinical, demographic, and socio-economic datasets to monitor and analyze COVID-19 infections, related hospitalizations and deaths [8]. Earlier Federal government policies to not track all COVID-19 breakthrough infections when they started to occur in the spring of 2021 [17,18] also contributed to the lack of U.S. population-based data to properly measure VE and possible waning of vaccine induced immunity.

To address these data gaps, we report on an observational study of over five million Medicare beneficiaries aged 65 and over who had completed their first series of COVID-19 vaccination with either the Pfizer-BioNTech BNT162b2 or the Moderna mRNA-1273 mRNA vaccines. This fully vaccinated older adult population belongs to a large Medicare population cohort of 17 million individuals who we had been monitoring for the U.S. Department of Defense (DoD) Joint Artificial Intelligence Center from the beginning of the pandemic through the Delta phase of the COVID-19 pandemic. As of September 2021, this large Medicare cohort included over 30% of the U.S. total COVID-19 related hospitalizations and over 50% of U.S. COVID-19 deaths, a consequence of the cohort’s higher risk profile for severe disease than the overall population, as previously reported [19]. 

We observed this large fully vaccinated older adult population during the Delta phase of the pandemic and reported this cohort’s rate of COVID-19 breakthrough infections, related hospitalizations, and deaths. In comparing their COVID-19 related outcomes with those of the total population cohort, we were able to measure COVID-19 VE and its evolution over time during this high morbidity Delta phase of the pandemic, analyzing the effect of time of vaccination, and variations in VE throughout the Delta phase surge. This study was based on a complete set of anonymized Medicare claim data received weekly for all individuals included in the cohort amounting to over 100 million claim lines a week, as well as socio-economic data obtained from the CDC Social Vulnerability Index (SVI) relating to our population cohort. The combination of these two data types allowed us to conduct our VE analyses and to statistically examine the relative importance of individual differences in clinical and socio-demographic factors leading to severe breakthrough infections.

As of October 2022, a year after the Delta surge, 71% of the U.S. 65 and over years old population have received their first COVID-19 vaccine booster dose while only 46% have received a second booster [13]. These low vaccine booster adoption rates call into question the likelihood of success of the campaign for the new bivalent boosters designed to provide protection against SARS-CoV-2 BA.4 and BA.5 Omicron subvariants. To support evidence-based COVID-19 vaccine booster policies, the ongoing monitoring of vaccine effectiveness over time in this highest risk population with the use of Medicare claim data at a large population scale is critically needed. We illustrate the type of needed monitoring and analytic approach in this reported study.

## 2. Materials and Methods 

### 2.1. Study Design and Data Sources

For this study, we, over 18 months, defined and observed a Medicare Fee-For-Service (FFS) beneficiary cohort of 20.5 million (M) individuals including 16.7 M individuals aged 65 and over who since 1 January 2020, either had a COVID-19 test (either positive or negative) or a COVID-19 diagnosis (identified by ICD-10-CM code U071 after 1 April 2020), or for any medical reason were hospitalized or had an emergency department, urgent care, or telehealth visit. As of August 2021, this Medicare cohort accounted for 6.2% of the total 2021 U.S. population, 30% of the 65 and over population and included 2.9M COVID-19 cases, 1.0 M COVID-19 hospitalizations and 341,000 COVID-19 related deaths between 1 March 2020, and 21 August 2021. Accounting for 7.7% of total U.S. COVID-19 cases [20], this higher risk cohort contributed over 50% of total U.S. COVID-19 deaths [21] and 30% of total COVID-19 related hospitalizations between July 2020 and August 2021 [22].

This study cohort was defined by Humetrix for Project Salus of the U.S. Department of Defense Joint Artificial Intelligence Center (JAIC) to monitor the COVID-19 pandemic and conduct predictive analytics to analyze and map COVID-19 infections, hospitalizations, and vaccinations to assist the military in their pandemic support mission. Under a Data Use Agreement between the Centers for Medicare and Medicaid Services (CMS) and the JAIC for Project Salus, de-identified outpatient and inpatient institutional Part A, Part B professional, skilled nursing facility (SNF), hospice, and Part D claims extending back to 1 October 2019, were extracted from the CMS Chronic Conditions Data Warehouse each week and transferred to a secure DoD network for processing and analysis by Humetrix. All direct identifiers of Medicare beneficiaries were removed from the claims data by CMS prior to data transfer. This study reports the results of an analysis of weekly claim files for the Salus Medicare cohort for the period 1 October 2019, through 17 September 2021. In addition to the clinical and demographic data generated from Medicare claims, the predictive models we developed used selected socio-economic variables from the CDC SVI through matching claim residential zip code and SVI Census Tract. 

### 2.2. Data Processing 

The Humetrix Extraction Transformation and Loading service (ETL) and analytics platform provided automated preprocessing of weekly updates of Medicare Part A inpatient and outpatient claims, hospice and SNF claims, Part B carrier claims and Part D prescription (PDE) claims to generate output files containing derived variables used to run the analyses as described in this paper. The Humetrix platform medical terminology service includes AMA CPT-4, FDA NDC, NPPES NPI, ICD-10-CM, CMS Level II HCPCS, and NLM RxNorm codes, and provides automated identification grouping of -10-CM codes to define chronic condition categories, and NDC drug code to RxNorm ingredient code mappings to identify pharmaceutical classes of active pharmaceutical ingredients (see Supplementary Materials).

### 2.3. Identification of COVID-19 Vaccinations, Breakthrough Infections and Breakthrough Hospitalizations

Vaccinations were identified by finding a CPT-4 code of 91301 for the Moderna mRNA-1273 vaccine or a CPT-4 code of 91300 for the Pfizer-BioNTech BNT162b2 vaccines in any claim. We excluded from this analysis the fewer number of beneficiaries that had been vaccinated with the Janssen COVID-19 vaccine. We also excluded from our analysis all the beneficiaries that received only one dose of vaccine or two doses of vaccine with a second dose too soon after the first dose, i.e., with less than 20 days between first dose and second dose for the Pfizer-BioNTech BNT162b2 vaccine or less than 27 days for the Moderna mRNA-1273 vaccine.

We defined a COVID-19 breakthrough infection as the first claim with an ICD-10-CM code of U07.1 occurring at least 14 days after the second dose of vaccine, without an ICD-10-CM code occurring between the first and second dose of the COVID-19 vaccine. This definition is consistent with the one used by the CDC [23].

We identified hospitalizations due to COVID-19 breakthrough infections based on: (1) an Inpatient claim with a primary admitting diagnosis ICD-10-CM code of U07.1 and a date of admission within 14 days of a breakthrough infection OR (2) any Inpatient claim with a date of discharge within 10 days of a subsequent breakthrough infection OR (3) a claim containing a place of service code of 21 (“in hospital”) and a date of service either 14 days after a breakthrough infection or 10 days before a breakthrough infection.

### 2.4. Imputation of COVID-19 Breakthrough Case and Hospitalization Counts

We observed that the Medicare claim-based vaccination rates in our older adult cohort represented on average 51% the vaccination rates for this age group published by the CDC for our observation time period of 1 July through 21 August 2021. These lower claim-based vaccination rates were expected, as many Medicare beneficiaries had received their vaccinations at mass vaccination sites for which no claims were submitted, resulting in missing vaccination data in Medicare claims. We corrected the lower claim-based breakthrough case counts in our cohort by using published CDC vaccination rates for the 65 and over population for that period of time [13]. We imputed breakthrough infection and hospitalization counts by multiplying the observed breakthrough case and hospitalization counts by the daily ratios of the CDC vaccination rate to our Medicare cohort vaccination rate (see Supplemental Materials). 

We estimated that imputed COVID-19 breakthrough case rates for the week ending 21 August 2021 (27 days prior to the last set of claims received by CMS on 17 September) were 83.2% (95% confidence interval 80.6–85.8%) of the case rates which would have been found if we had continued to receive CMS claims data 90 days or more after 21 August 2021 (see Supplementary Materials Figure S1). For breakthrough hospitalization rates, we used a cutoff of the week ending 14 August 2021 in view of the median seven-day lag between the onset of COVID-19 symptoms and hospitalization [24,25].

### 2.5. Estimation of mRNA Vaccine Effectiveness

Vaccine Effectiveness of the Moderna mRNA-1273 vaccine and the Pfizer-BioNTech BNT162b2 vaccine were estimated using the VE screening method [26] and a fitting by logistic regression according to Farrington [27,28]. The fitting procedure enabled us first to obtain 95% confidence intervals and second to introduce calendar week number as the only covariate of the model. The calendar week number was expanded with linear tail-restricted cubic spline function to get a smoothened evolution of the VE over time [29]. The logistic regression was performed with R statistical software, version 3.6 with the rms package [30,31]. VEs were estimated for the period going from 10 July 2021 to 21 August 2021, when the peak of the Delta phase of the pandemic was reached. At that time, the CDC reported vaccination rates for the 65 and over population remained constant around 80%.

### 2.6. Vaccination Time Dependence of Breakthrough Outcomes

We defined two groups of vaccinated beneficiaries from our Medicare cohort: those who received a second dose of vaccine between 18 January 2021, and 31 January 2021 (“January Group”), and those who received a second dose of vaccine between 8 March 2021, and 21 March 2021 (“March Group”). We compared the weekly COVID-19 breakthrough infection and hospitalization rates between these two groups (stratified by age group and by specific vaccine) during the Delta phase of the pandemic from 19 June 2021 to 21 August 2021.

### 2.7. Logistic Regression Model to Identify Predictors of COVID-19 Breakthrough Hospitalization

As in previously published risk analysis for COVID-19 hospitalization prior to the approval of COVID-19 vaccines [19], we used logistic regression to identify significant predictors of COVID-19 breakthrough hospitalizations using R statistical software, version 3.6 with the rms, glmnet, and pROC packages [30,31,32,33] prior to the SARS CoV-2 Delta variant phase of the pandemic [34] (see Supplemental Materials).

## 3. Results

### 3.1. Study Population Characteristics

The seven terabyte Medicare cohort database collected during the tenure of Project Salus included all inpatient (Part A), outpatient institutional and carrier (Part B), skilled nursing facility, hospice, and PDE (Pharmacy) Medicare claims dating back to 1 October 2019, for 20.5 M beneficiaries enrolled in fee-for-service (FFS) Medicare. From 1 March 2020 through 21 August 2021, this cohort accounted for 7.7% of total U.S. COVID-19 cases [19], over 50% of total U.S. COVID-19 deaths [20] and 30% of total COVID-19 related hospitalizations between July 2020 and August 2021 [21]. For this Vaccine Effectiveness study, we elected to restrict our VE analysis to the 16.5M Medicare beneficiaries in this cohort who were 65 or older years old to enable us to compare our VE results with other published data on this age group. As shown in Table 1, from December 2020 through August 2021, we identified 5.6 M beneficiaries who had been fully vaccinated with either the Pfizer-BioNTech BNT162b2 mRNA vaccine (n = 2.7 M) or the Moderna mRNA-1273 vaccine (n = 2.9 M). The distribution of vaccinees in the cohort by date of full vaccination is shown in Figure S2 in the Supplementary Materials; the median day for full vaccination of the cohort was 8 March 2021 (IQR 18 February 2021, to 30 March 2021), suggesting that our “March Group” was selected near the midpoint of the initial vaccination program. From January through August, the cumulative percentage of fully vaccinated beneficiaries with a breakthrough COVID-19 infection rose to 2.6% (n = 146,189), of whom 21% required hospitalization (n = 30,454).

Detailed characteristics of the FFS Medicare Salus cohort used in this observational study are shown in Table 1. This cohort composed of beneficiaries 65 and over years old with a median age of 76 years old (IQR 71–82). Compared to the 65 and over general Medicare population [35] the age distribution is weighted toward the older age categories (45% vs. 57% in the 65–74 age group, 36% vs. 30% in the 75–84 age group, 19% vs. 13% in the 85 and over group). Of the cohort, 57% were female, 12.7% were disabled, 10.2% lived in zip codes in the lowest national income quartile, and 15.9% were dually enrolled in Medicare and Medicaid programs. The rates of chronic conditions in this cohort are also higher than in the overall 65-year and over Medicare population (chronic kidney disease 35.2% vs. 24.9%; COPD 24.3% vs. 11.4%; CHF 24.1% vs. 14.6%; Diabetes 36.2% vs. 27.1%; ischemic heart disease 44.3% vs. 28.6%; asthma 14% vs. 4.5%) [36].

Of the 5.58 M beneficiaries who were fully vaccinated, 2.6% had a breakthrough infection (n = 146,189) observed through 21 August 2021, 20.8% of which required hospitalization for COVID-19 (n = 30,454). We found that age, sex, and race variables, presence or absence of prior COVID-19, presence or absence of prior hospitalization and presence or absence of obesity or morbid obesity were all significantly associated with hospitalization in breakthrough infections (Chi-square tests; all *p* values < 0.001). With regard to risk factors for hospitalization in COVID-19 breakthrough infections, univariate analysis revealed that beneficiaries with the highest case hospitalization rates were those with the following comorbidities: ESRD (41%), an organ transplant (35%), leukemia (33%), pulmonary fibrosis or pulmonary hypertension (33%), lung cancer (31%), and chronic liver disease (31%). Beneficiaries with a prior hospitalization for any reason also had a high case hospitalization rate at 29%. Beneficiaries with the lowest case hospitalization rates were those with a case of COVID-19 prior to vaccination (13%), and those in the 65–74 age group (18.4%).

### 3.2. Evolution of COVID-19 mRNA Vaccine Effectiveness during the Delta Phase

As described in the Materials and Methods section, we imputed daily number of COVID-19 breakthrough infections and hospitalizations using the CDC reported daily fully vaccinated rates for the 65 and over years old U.S. population [13] as shown in Tables S1 and S2 in Supplementary Materials. We monitored these imputed weekly COVID-19 breakthrough infection rates and hospitalization rates in vaccinated and unvaccinated groups of beneficiaries. in the July–August time period as the SARS-CoV-2 Delta variant became dominant. During this time period of 10 July 2021 through 21 August 2021, as the SARS-CoV-2 Delta variant increased in prevalence from about 82% to 99% [37] there were 181,569 new COVID-19 cases, with 117,411 imputed breakthrough infections in fully vaccinated beneficiaries with 23,164 breakthrough case hospitalizations occurring in fully vaccinated beneficiaries (Tables S1 and S2 in Supplementary Materials).

**Figure 1:**
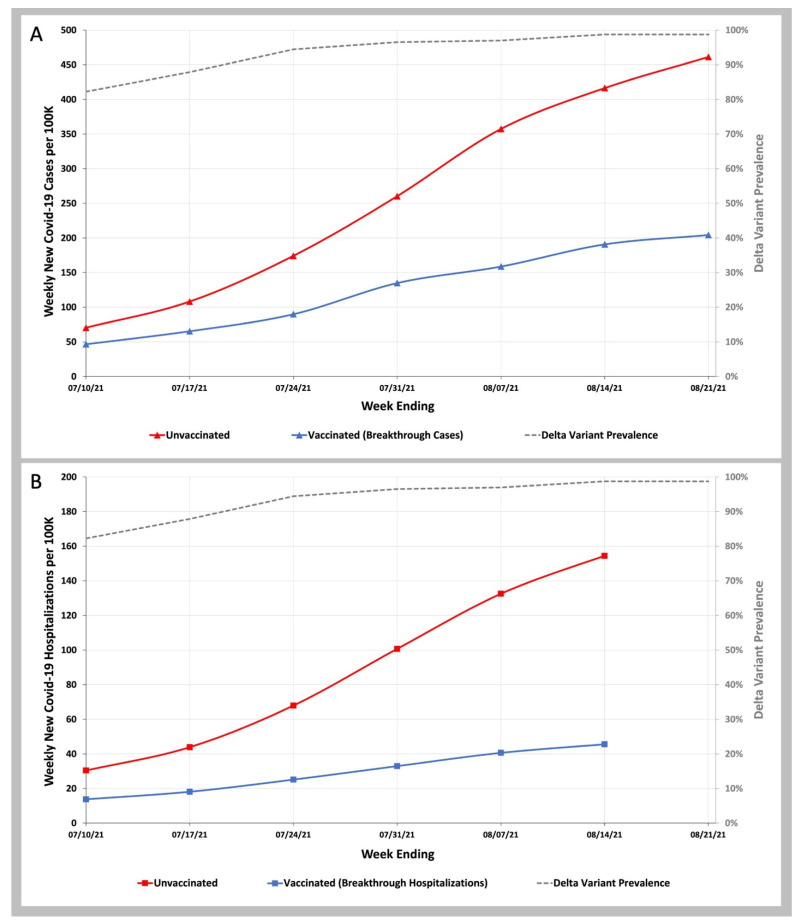
COVID-19 Infection and Hospitalization Rates in Vaccinated and Unvaccinated Individuals during the Delta Variant Phase of the Pandemic. (**A**)—Weekly infection rates per 100 K vaccinated (blue line) and unvaccinated (red line) individuals. (**B**)—Weekly hospitalization rates per 100 K vaccinated (blue line) and unvaccinated (red line) individuals. Weekly SARS-CoV-2 Delta variant prevalence reported by the CDC (gray dashed line).

We compared the rate of COVID-19 infection in vaccinated beneficiaries and the imputed rate of COVID-19 infection in unvaccinated beneficiaries and found that unvaccinated beneficiaries were 1.5 times more likely to become infected than vaccinated beneficiaries in the week ending 10 July 2021, and by 21 August 2021, these unvaccinated beneficiaries were 2.3 times more likely to be infected than vaccinated beneficiaries (Figure 1A and Table S3 in Supplementary Materials). In comparing COVID-19 hospitalizations in vaccinated and unvaccinated beneficiaries between the week ending 10 July 2021, and the week ending 14 August 2021, the ratio of COVID-19 breakthrough hospitalizations in unvaccinated beneficiaries to vaccinated beneficiaries rose from 2.2 to 3.4-fold (Figure 1B and Table S4 in Supplementary Materials).

**Figure 2:**
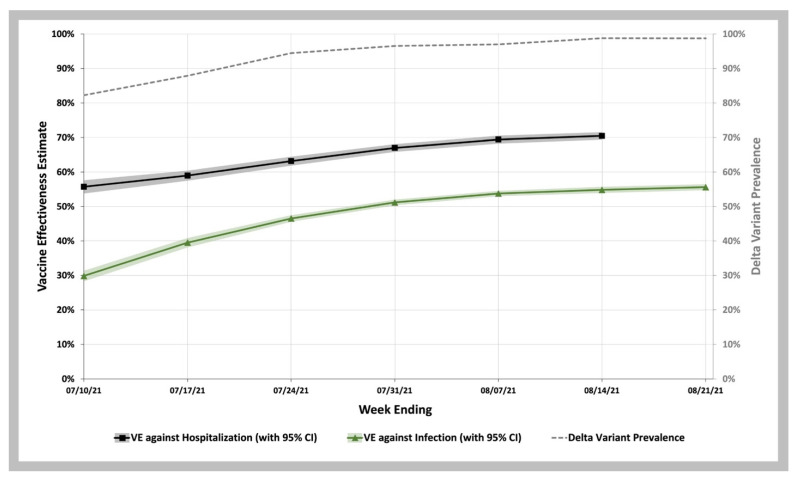
COVID-19 Vaccine Effectiveness against Infection and Hospitalization during the Delta Variant Phase of the Pandemic. Vaccine effectiveness estimates against infection (green curve with 95% CI shown in green shaded area) and against hospitalization (black curve with 95% CI shown in gray shaded area). Weekly SARS-CoV-2 Delta variant prevalence reported by the CDC (gray dashed line).

To estimate vaccine effectiveness against COVID-19 infection and hospitalization, we used the VE screening method of Farrington [27,28,29]. As shown in Figure 2, we observed that between the week ending 10 July and 21 August 2021, when the Delta prevalence remained high ranging from 83% to 99%, VE increased over this time, from 29.8% (95% C.I. 28.3–31.4%) to 55.6% (95% C.I. 54.7–56.5%). Between the week ending 10 July and 14 August 2021, VE against COVID-19 hospitalization increased from 55.7% (95% C.I. 53.7–57.6%) to 70.5% (95% C.I. 69.4–71.5%) (Tables S7 and S8 in Supplemental Materials).

### 3.3. Differences in COVID-19 Breakthrough Infection and Hospitalization Rates between Beneficiaries Fully Vaccinated in January 2021 Group and March 2021 Group and by mRNA Vaccine Type

**Figure 3:**
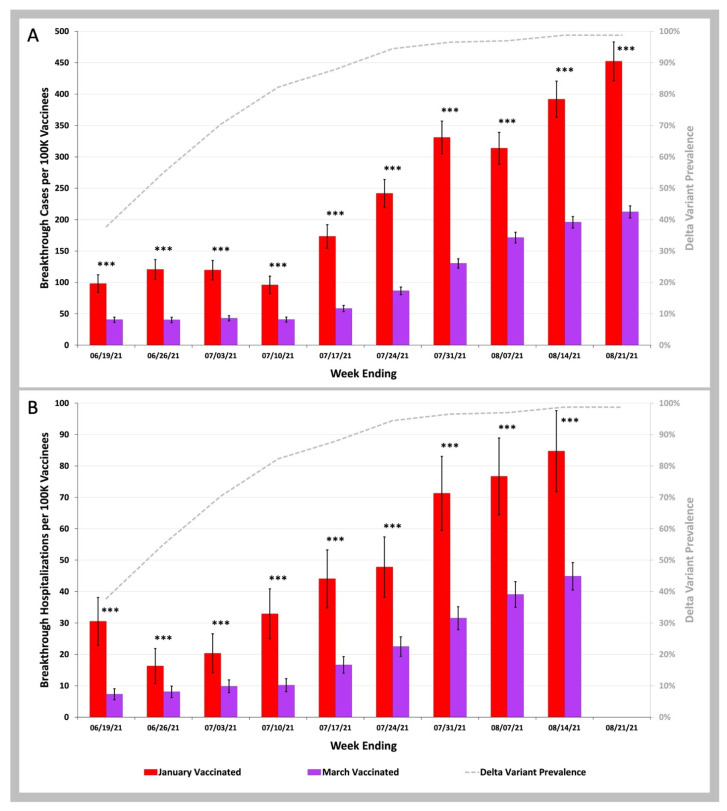
COVID-19 Breakthrough Infection and Hospitalization Rates in Individuals Vaccinated in January versus March 2021. (**A**)—Weekly infection rates per 100 K individuals vaccinated in January 2021 (red bars) vs. vaccinated in March 2021 (purple bars). (**B**)—Weekly hospitalization rates per 100 K individuals vaccinated in January 2021 (red bars) vs. vaccinated in March 2021 (purple bars). Weekly SARS-CoV-2 variant prevalence reported by the CDC (gray dashed line). Error bars indicate 95% CI. Asterisks shown above the bars indicate p values for weekly rates for those vaccinated in January 2021 being greater than weekly rates for those vaccinated in March 2021. *** *p* < 0.001 by two-proportion Z-test.

In Figure 3 we compared the COVID-19 infection and COVID-19 hospitalization rates during the SARS-CoV-2 Delta variant wave from June to August 2021 for two groups of beneficiaries: those who received their second dose of vaccine in a 2-week period in January 2021 (18–31 January) versus those who received their second dose of vaccine during a 2-week period in March 2021 (8–21 March). For both COVID-19 breakthrough infection rates and hospitalization rates, the January Group of vaccinees (red bars) had higher rates than the March Group (purple bars; *p* values < 0.001 for every comparison). In further analysis we found that the COVID-19 infection rates for the January Group were nearly identical to the imputed rates seen in unvaccinated beneficiaries (Table S5 in Supplemental Materials). For COVID-19 breakthrough infection hospitalization rates, (Table S6 in Supplementary Materials) we found that the January Group had 1.38 (95% CI 1.09–1.68) lower rates than unvaccinated beneficiaries. There was much better protection against hospitalization for the March Group who had 3.11 (95% CI 2.87–3.35) lower breakthrough hospitalization rates then unvaccinated beneficiaries.

**Figure 4:**
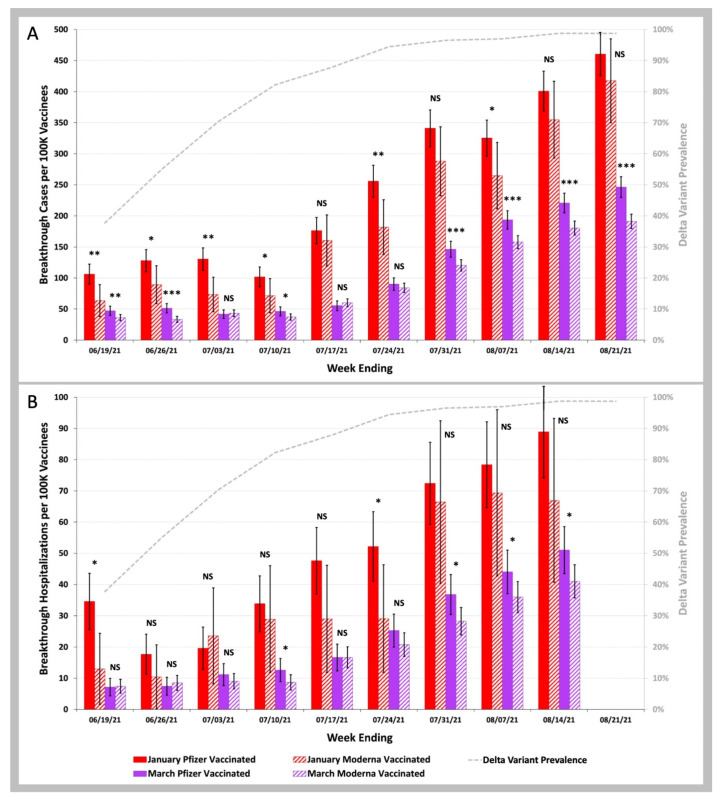
COVID-19 breakthrough infection and hospitalization rates in individuals vaccinated in the January Group vs. those vaccinated in the March Group with either the Pfizer-BioNTech BNT162b2 or the Moderna mRNA-1273 vaccine. (**A**)—Weekly infection rates per 100 K individuals vaccinated in the January Group (red bars) vs. those vaccinated in the March Group (purple bars), with either the Pfizer-BioNTech BNT162b2 (solid bars) or the Moderna mRNA-1273 (striped bars) vaccines. (**B**)—Weekly hospitalization rates per 100 K individuals vaccinated in the January Group (red bars) vs. those vaccinated in the March Group (purple bars), with either the Pfizer-BioNTech BNT162b2 (solid bars) or the Moderna mRNA-1273 (striped bars) vaccine. Weekly SARS-CoV-2 Delta variant prevalence reported by the CDC (gray dashed line). Error bars indicate 95% CI. Symbols shown above the red bars (January 2021 vaccinees) or purple bars (March 2021 vaccinees) indicate p values for weekly rates for those vaccinated with the Pfizer-BioNTech BNT162b2 vaccine being greater than weekly rates for those vaccinated with the Moderna mRNA-1273 vaccine. NS *p* ≥ 0.05; * *p* < 0.05; ** *p* < 0.01; *** *p* < 0.001 by two-proportion Z-test.

In Figure 4, we plotted the COVID-19 breakthrough infection and hospitalization rates for the January versus March Groups for those who received the Pfizer-BioNTech BNT162b2 vaccine (January Group as the solid red bars, March Group as the solid purple bars) and the Moderna mRNA-1273 vaccine (January Group as the striped red bars, March Group as the striped purple bars). For the weeks ending after 24 July 2021, the COVID-19 breakthrough infection and hospitalization rates in the March Group were significantly lower for beneficiaries vaccinated with the Moderna mRNA-1273 vaccine than with the Pfizer-BioNTech BNT162b2 vaccine: *p* < 0.001 for Pfizer vs. Moderna for COVID-19 breakthrough infections; *p* < 0.05 for Pfizer vs. Moderna for COVID-19 breakthrough hospitalizations.

### 3.4. Differences in Breakthrough Infections and Hospitalizations by Age

To examine whether beneficiaries age impacted the observed differences in breakthrough infections and hospitalizations, we next compared the age distribution of beneficiaries who completed their primary vaccination series in January 2021 versus those that were vaccinated in March 2021 (Table 2). We found that, as expected given that the oldest individuals and nursing home residents were first prioritized for vaccination, in comparison to January vaccinees, the March vaccinees showed a large decrease in the oldest age group of individuals aged 85 and above from 42% to 14%.

**Figure 5:**
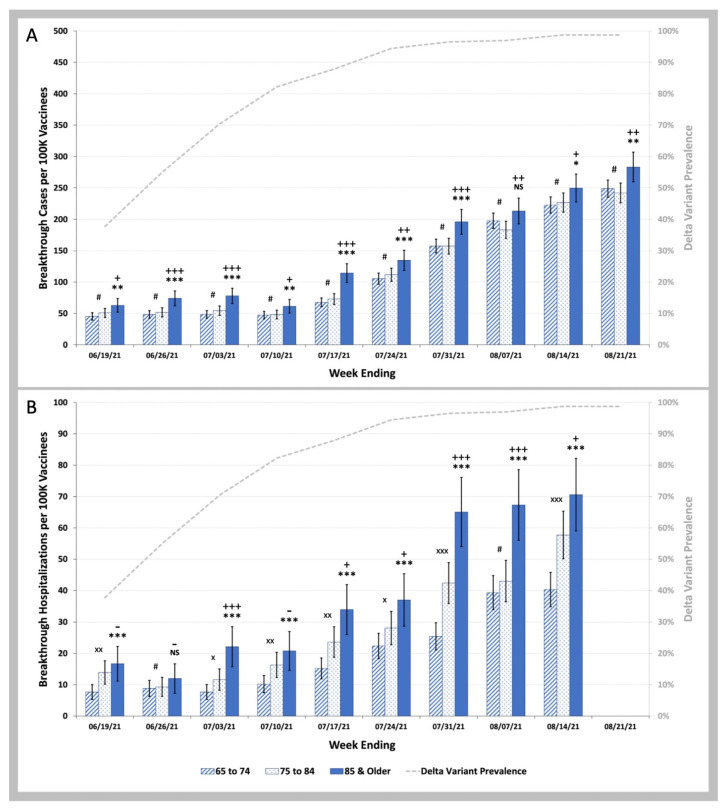
COVID-19 Breakthrough Infection and Hospitalization Rates in Individuals Vaccinated in the January Group, and in the March Group by Age. (**A**)—Weekly infection rates per 100 K individuals vaccinated in the January 2021 and March 2021 vaccinee groups aged 65 to 74 (striped blue bars) vs. aged 75 to 84 (stippled blue bars) vs. aged 85 and older (solid blue bars). (**B**)—Weekly hospitalization rates per 100 K individuals vaccinated in January 2021 and March 2021 aged 65 to 74 (striped blue bars) vs. aged 75 to 84 (stippled blue bars) vs. aged 85 and older (solid blue bars). Weekly SARS-CoV-2 Delta variant prevalence reported by the CDC (gray dashed line). Error bars indicate 95% CI. Symbols shown above the bars indicate p values for weekly rates for the vaccinees aged 75 to 84 being greater than weekly rates for the vaccinees aged 65 to 74, *p* values for weekly rates for the vaccinees aged 85 and over being greater than weekly rates for the vaccinees aged 65 to 74, and *p* values for weekly rates for the vaccinees aged 85 and over being greater than weekly rates for the vaccinees aged 75 to 84. # *p* ≥ 0.05; × *p* < 0.05; ×× *p* < 0.01; ××× *p* < 0.001 by two-proportion Z-test (rate for 75 to 84 greater than rate for 65 to 74). NS *p* ≥ 0.05; * *p* < 0.05; ** *p* < 0.01; *** *p* < 0.001 by two-proportion Z-test (rate for 85 and older greater than rate for 65 to 74). - *p* ≥ 0.05; + *p* < 0.05; ++ *p* < 0.01; +++ *p* < 0.001 by two-proportion Z-test (rate for 85 and older greater than rate for 75 to 84).

We then examined whether the difference in age could explain the observed differences in COVID-19 breakthrough infection and hospitalization rates for beneficiaries vaccinated in the January Group versus the March Group. There were consistent statistically significant increases in COVID-19 breakthrough infection rates during the Delta phase of the pandemic for the 85 and over years old beneficiaries compared to the 65 to 74 years old beneficiaries (Figure 5A). Although the difference in these rates were far less than the 2.50 fold (95% CI 2.23–2.74), differences in COVID-19 breakthrough infection rates between the January and March Groups are shown in Figure 3A (and Table S5 in Supplemental Materials). For COVID-19 breakthrough hospitalization rates (Figure 5B), there were highly significant differences in rates for the 85 and over year old beneficiaries compared to the 65–74 years old beneficiaries suggesting that the age differences shown in Table 2 might account for the 2.50-fold (95% C.I. 1.99–2.98) difference in hospitalization rates between January and March Groups shown in Figure 3B (and Table S6 in Supplemental Materials).

### 3.5. Analytic Model to Identify Individual Predictors of COVID-19 Breakthrough Hospitalization

We used logistic regression to identify risk factors for COVID-19 hospitalizations in breakthrough infections which occurred between 6 February and 10 July 2021. Based on the published prevalence of SARS-CoV-2 genomic variants [34], we estimate that 90% of COVID-19 cases during this time period were caused by variants which preceded the Delta B.1.617.2 variant (see Supplemental Methods Section 1.9 in Supplementary Materials). The model achieved an Area Under the Receiver Operating Characteristics (AUROC) curve of 0.73 and a balanced accuracy of 0.67 using a 0.50 threshold.

**Figure 6:**
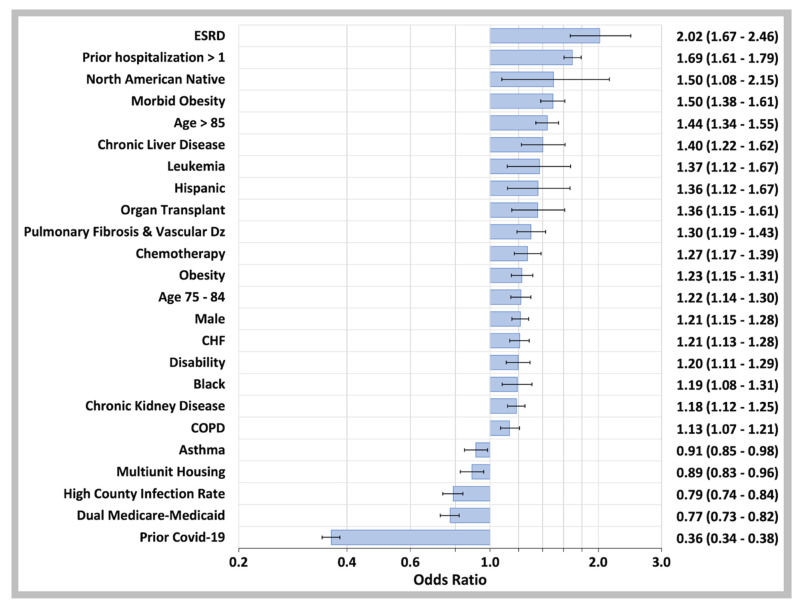
Predictor Variables for COVID-19 Breakthrough Case Hospitalization. Odds ratios determined by binary logistic regression analysis of confirmed COVID-19 breakthrough cases that required hospitalization for the disease and those that were managed with outpatient care only. The independent variables are on the left of the plot while the odds ratios values with 95% CI are on the right. Not shown: anticoagulant drugs (OR 1.20, 95% CI 1.12–1.29), opioid drugs (OR 1.20, 95% CI 1.11–1.30), antiplatelet drugs (OR 1.17, 95% CI 1.07–1.28), NSAID drugs (OR 1.14, 95% CI 1.03–1.27), Beta-2 agonist drugs (OR 1.12, 95% CI 1.02–1.23), ACE inhibitor drugs (OR 1.09, 95% CI 1.03–1.16), immunosuppressive drugs (OR 0.89, 95% CI 0.79–0.99), Azithromycin drugs (OR 0.88, 95% CI 0.77–1.00), or other ethnicities (OR 0.76, 95% CI 0.61–0.97). Multiunit housing variable: CDC SVI_EPL_MUNIT top quartile; High County Infection rate: beneficiary county of residence in the top quartile of U.S. county infection rate 14 days prior to COVID-19 diagnosis date.

As shown in Figure 6, the top five predictors of COVID-19 hospitalization in breakthrough infections were ESRD, having more than one prior hospitalization (for any reason), North American Native race, age 85 and older, and morbid obesity with other important risk factors including chronic liver disease, leukemia, Hispanic ethnicity, and a history of organ transplantation. COVID-19 infection prior to vaccination had a major protective effect against hospitalization for COVID-19 breakthrough infections (OR 0.36, 95% CI 0.34–0.38). The variables which did not survive backward variable selection using the Akaike Information Criterion (AIC) to remove non-significant variables are listed in the Supplementary Materials.

The model also included a linear effect continuous variable of the number of days between full vaccination and COVID-19 breakthrough infections. Using the logistic regression coefficient for this variable of 0.0055 (95% CI 0.0046–0.0063; *p* < 0.001) to illustrate the potential magnitude of its effect in the model, the calculated adjusted odds ratio for breakthrough hospitalization for an individual vaccinated 140 days earlier (the number of days between the median day of full COVID-19 vaccination for the 65 and over years old cohort and the midpoint of the weeks shown in Figure 3B) would be 2.15 (95% CI 1.90–2.41).

## 4. Discussion

This is a real-world study monitoring Vaccine Effectiveness (VE) of the Pfizer-BioNTech BNT162b2 and Moderna mRNA-1273 mRNA vaccines on a weekly basis during the SARS-CoV-2 Delta B.1.617 variant phase of the COVID-19 pandemic in a 65 years and older Medicare population cohort at higher risk for severe COVID-19 infection [19]. In this cohort with a much higher prevalence of comorbidities and other risk factors for severe COVID-19 than in the overall Medicare population, we found that VE increased as the Delta phase progressed. Specifically, as the SARS-CoV2 variant prevalence rose from 82% to 89% between 10 July and 21 August 2021, VE against infection increased from 30% to 56%, and VE against COVID-19 hospitalization increased from 56% to 71%. Compared with published reports of the mRNA vaccine VE [38] during the Delta pandemic phase in different populations and countries, our estimates of VE against COVID-19 infection and hospitalization due to the SARS-CoV-2 Delta variant fall within the lower end of reported ranges, similar to the 43% VE against infection in a U.S. nursing home population [36], and also at the low end of the mRNA vaccine VE estimates of 70% to over 90% against hospitalization [38,39,40]. These lower VE rates are likely explained by the older age distribution of our cohort resulting in reduced immune responses to COVID-19 vaccines [41,42,43] and the higher rates of comorbidities of our selected Medicare cohort. These individual characteristics can be explained by our cohort selection criteria enrolling Medicare beneficiaries using healthcare services for any reason and not limited to only those with a COVID-19 diagnosis.

Looking at the time period of vaccination, we observed that COVID-19 case rates and hospitalizations were 2.5 fold higher during the period of Delta variant predominance for Medicare beneficiaries fully vaccinated in January 2021 versus those fully vaccinated in March 2021, a result similar to that reported in Israel [11]. While a major difference in the January and March Groups was the much higher proportion of 85 and older individuals in the January vaccinees, this age difference did not account for the difference in observed COVID-19 infection rates. We believe that waning immunity most likely accounts for the much higher COVID-19 case rates through July and August for the January Group compared to the March Group. This conclusion is also supported by numerous publications documenting significant waning immunity four to five months after full vaccination [38,39,40] and our multivariate logistic regression model showing an important effect of the number of days since full vaccination and the risk of hospitalization in COVID-19 breakthrough infections during the Alpha phase of the pandemic.

Our cohort included a near equal number of individuals fully vaccinated with the Pfizer-BioNTech BNT162b2 (n = 2.7 M) and the Moderna mRNA-1273 vaccines (n = 2.9 M) which enabled us to compare the performance of these two vaccines during the Delta phase of the pandemic. We found that for those individuals fully vaccinated in the March Group, the Moderna mRNA-1273 vaccine provided better protection against COVID-19 infection during the Delta phase of the pandemic than the Pfizer-BioNTech BNT162b2 vaccine, confirming a previously published report published by the UK Health Security Agency [44].

Looking at risk factors for COVID-19 breakthrough hospitalizations, we trained and validated a logistic regression model including individual demographics, socioeconomic factors, co-morbidities, prior health care utilization, prior COVID-19 infection, and time of vaccination during the phase of the pandemic before the Delta variant accounted for more than 10% of COVID-19 cases. The most prominent risk factors we found were: ESRD, age of 85 and over, one or more prior hospitalizations since 2019, and the number of days since full COVID-19 vaccination. This last finding strongly supports that waning immunity plays a significant role in the risk of developing a severe case of a COVID-19 breakthrough infection requiring hospitalization. This model also demonstrated an important protective effect of prior COVID-19 infection in preventing severe breakthrough infections. This result adds to a growing number of published reports that prior COVID-19 infection significantly boosts vaccine effectiveness [45] and postpones waning immunity [46] consistent with published reports that prior infection is associated with higher levels of immune activation following COVID-19 vaccination [47].

Given that we observed waning immunity in this cohort, what could explain this unexpected and not previously reported finding of an increase in VE during the Delta phase of the pandemic? It is noteworthy that just prior to the Delta surge, the CDC had issued a new guidance on 13 May 2021 advising that fully vaccinated individuals no longer needed to wear masks or practice social distancing [48]. Then, once the Delta wave took hold in July 2021 with large increases in COVID-19 cases nationwide, the CDC and the White House reversed course on 30 July 2021 advising everyone to wear masks indoors regardless of their vaccination status [49,50]. One potential reason for increasing VE through July and August as the Delta phase of the pandemic intensified with case counts rising from 11,000 a day on 20 June to 166,000 a day on 1 September 2021 [51] is that individuals adopted increased mask wearing which has been shown to lower risk of infection with the Delta variant [52] as well as social distancing in order to lower their chances of COVID-19 infection starting even before the federal government guidelines changed. The importance of policy and individual behavioral changes in mask wearing and social distancing in producing major changes in VE during this time period has been recognized by others [40], and likely accounts for the markedly lower mortality due to COVID-19 in Japan compared to the United States [53]. Our results support the conclusion that public adoption of these prevention policies likely accounts for the increasing VE against infection as we found a much greater relative VE increase of 87% against COVID-19 infection but only a relative increase of 27% VE increase against COVID-19 hospitalization.

## 5. Limitations

The lack of laboratory confirmation of SARS-CoV-2 infection in claims data prevents the detection of asymptomatic cases and could result in COVID-Like Illnesses (CLI) being misdiagnosed as COVID-19 cases. Secondly, the claim reported vaccination rate for this cohort was only 51% of the rate reported by the CDC for the 65 and over US population during the period of this study as a result of many Medicare beneficiaries receiving vaccinations at mass vaccination sites that did not submit vaccination claims to CMS. Our method of imputing the number of COVID-19 breakthrough cases and hospitalization by using the ratio of the CDC over 65 vaccination rate and the vaccination rate identified through claims may have resulted in an underestimate of VE. However, the population of participants in our study is older than the overall 65 and over U.S. population, and should be even more highly vaccinated. As a result, our method may have led us to underestimate COVID-19 breakthrough infections and hospitalizations and overestimate VE. The absence of accurate vaccination data also prevented us from matching beneficiaries who had been vaccinated with those who had not been vaccinated for age, sex, race, location, and co-morbidities, which would have enabled us to more accurately estimate VE against COVID-19 infection and hospitalization. The screening method [26,27,28,29] we used for estimating VE is not the most accurate method of assessing VE and may in part account for why our VE estimates differ from published VE results during the SARS-CoV-2 Delta variant surge [38,54]. We consider that the most likely explanation for our lower VE estimates though is due to the higher risk profile of our older adult cohort with a higher burden of comorbidities. Lastly, the hypothesis that individual preventive behaviors including social distancing and mask wearing might account for increasing vaccine effectiveness during the Delta surge could not be tested due to lack of individual behavior data for Medicare beneficiaries. Collecting such behavioral data should be a priority for future vaccine effectiveness studies in this higher risk population for whom a multilayer prevention approach is essential.

## 6. Conclusions

This real world COVID-19 vaccine effectiveness study shows evolving vaccine effectiveness with both waning vaccine-induced immunity and increasing vaccine effectiveness as the Delta surge progressed. The large majority of this study cohort of 65 and over year old Medicare beneficiaries at highest risk for severe COVID-19 was fully vaccinated well before the Delta phase of the pandemic and clearly benefited from vaccination with reduced rates of infection and especially reduced rates of severe disease. However, they still experienced a 2.6% cumulative rate of breakthrough infections through August 2021 which is more than double the 1.15% breakthrough rate previously reported [55]. As of November 2022, only 27% of the 65 and over U.S. population has received a bivalent vaccine booster dose [13,56,57,58] designed to provide protection against the more infectious Omicron BA.4.6 and BA.5 Omicron subvariants accounting for 80% of the circulating strains of SARS-CoV-2 in the U.S. [59]. In view of this poor booster adoption, our findings of enhanced vaccine effectiveness during the Delta phase of the pandemic likely due to increased masking and social distancing strongly support the need for a multilayered prevention approach.

The large Medicare study cohort we assembled which accounted for over 50% of total U.S. COVID-19 related deaths from March 2020 through August 2021 provides an important population cohort for ongoing pandemic monitoring to optimize public health policies that protect these higher risk individuals through COVID-19 booster vaccination and other preventive measures such as masking. In the face of this unrelenting pandemic, ongoing monitoring of the Medicare population is more than ever needed as previously recognized [60,61]. The Medicare claim dataset and analytics platform used for this study can serve as a template to conduct pandemic monitoring, serial monitoring of COVID-19 vaccine and antiviral drug effectiveness. This would complement existing and newly launched CDC disease surveillance systems [62,63,64] at a time when the CDC has embarked on a comprehensive set of reforms to upgrade its disease forecasting and program evaluation performance, as well as help the FDA address major data gaps it has faced when approving COVID-19 vaccines and therapeutics and in post-marketing surveillance of these products [65].

## Figures and Tables

**Table 1:** Study Cohort: Demographic, Clinical and Socioeconomic Characteristics.

	Medicare Cohort (65 and Over)		Fully Vaccinated ^†^		Breakthrough Cases		Breakthrough Not Hospitalized ^‡^		Breakthrough Hospitalized ^¶^		
Variable	Value (%) ^1^	Count	Value (%) ^2^	Count	Value (%) ^3^	Count	Value (%) ^4^	Count	Value (%) ^4^	Count	Flag
Total Study Population	100%	16,679,436	33.5%	5,583,759	2.6%	146,189	79.2%	115,735	20.8%	30,454	
Age (median)		**76**		**76**		**77**		**77**		**78**	
Age (IQR)		71–82		71–82		72–85		71–85		72–86	+++
Study Population Age 65–74	45.1%	7,519,583	33.3%	2,500,523	2.3%	57,264	81.6%	46,703	18.4%	10,561	***
Study Population Age 75–84	35.8%	5,970,510	33.9%	2,024,853	2.5%	50,979	78.1%	39,795	21.9%	11,184	***
Study Population Age 85+	19.1%	3,189,343	33.2%	1,058,383	3.6%	37,946	77.0%	29,237	23.0%	8709	***
Male	43.1%	7,181,793	31.8%	2,283,212	2.6%	59,015	76.9%	45,401	23.1%	13,614	***
Female	56.9%	9,497,643	34.8%	3,300,547	2.6%	87,174	80.7%	70,334	19.3%	16,840	***
North American Native	0.5%	88,219	32.3%	28,509	2.8%	812	74.3%	603	25.7%	209	***
Black	8.0%	1,329,525	28.0%	372,916	3.1%	11,470	76.9%	8816	23.1%	2654	***
Hispanic	1.7%	289,452	25.1%	72,619	3.2%	2298	77.9%	1791	22.1%	507	
Asian	2.1%	347,900	34.2%	119,041	2.1%	2447	81.4%	1993	18.6%	454	**
White	83.8%	13,985,425	34.1%	4,762,386	2.6%	124,521	79.3%	98,704	20.7%	25,817	
Lowest Income (EPL_PCI)	10.2%	1,709,397	26.9%	460,182	3.3%	15,388	77.8%	11,972	22.2%	3416	***
Most Crowded Housing (EPL_CROWD)	9.9%	1,656,673	28.7%	475,836	3.0%	14,338	80.1%	11,479	19.9%	2859	**
Highest Multi-unit Housing (EPL_MUNIT)	11.7%	1,959,686	34.0%	666,470	2.9%	19,097	80.5%	15,375	19.5%	3722	***
Highest Institutional Housing (EPL_GROUPQ)	7.4%	1,226,017	31.8%	389,673	2.8%	10,756	80.1%	8618	19.9%	2138	*
Medicare Status: Disabled	12.7%	2,115,452	30.5%	645,221	3.4%	21,694	77.5%	16,810	22.5%	4884	***
Dual Medicare-Medicaid	15.9%	2,657,760	31.0%	824,903	5.2%	42,757	82.4%	35,238	17.6%	7519	***
Prior Hospitalization: 1 or more ^#^	N/A	N/A	N/A	N/A	N/A	54,936	70.6%	38,799	29.4%	16,137	***
COVID-19 prior to vaccination ^##^	N/A	N/A	N/A	708,159	8.8%	62,026	86.7%	53,790	13.3%	8236	***
Comorbidities: ESRD	1.1%	189,253	27.6%	52,201	4.4%	2280	59.5%	1357	40.5%	923	***
Comorbidities: Chronic Kidney Disease	35.2%	5,863,413	32.5%	1,905,160	3.1%	59,615	75.3%	44,906	24.7%	14,709	***
Comorbidities: Pulmonary Fibrosis HTN	8.0%	1,341,591	33.3%	447,216	3.1%	13,906	67.1%	9326	32.9%	4580	***
Comorbidities: Chronic Liver Disease	2.7%	456,512	32.0%	145,869	3.6%	5180	69.1%	3579	30.9%	1601	***
Comorbidities: COPD	24.3%	4,051,522	31.5%	1,277,736	3.4%	43,259	75.5%	32,651	24.5%	10,608	***
Comorbidities: CHF	24.1%	4,013,279	31.4%	1,261,429	3.6%	45,411	74.8%	33,985	25.2%	11,426	***
Comorbidities: Stroke/TIA	13.7%	2,289,116	32.5%	742,873	3.7%	27,345	77.5%	21,185	22.5%	6160	***
Comorbidities: Diabetes	36.2%	6,033,373	32.5%	1,958,212	3.1%	60,984	76.8%	46,865	23.2%	14,119	***
Comorbidities: Hypertension	75.8%	12,640,608	33.6%	4,249,768	2.8%	116,996	78.2%	91,443	21.8%	25,553	***
Comorbidities: History of Acute MI	0.9%	146,296	28.9%	42,287	3.1%	1328	72.1%	957	27.9%	371	***
Comorbidities: Ischemic Heart Disease	44.3%	7,387,973	32.6%	2,408,026	3.1%	73,977	77.0%	56,984	23.0%	16,993	***
Comorbidities: Asthma	14.0%	2,337,318	33.6%	785,490	3.0%	23,920	77.1%	18,438	22.9%	5482	***
Comorbidities: Chemotherapy	10.7%	1,777,575	35.6%	633,198	4.1%	26,224	73.7%	19,331	26.3%	6893	***
Comorbidities: Obesity	17.4%	2,901,845	35.8%	1,040,249	3.0%	31,074	76.8%	23,876	23.2%	7198	***
Comorbidities: Morbid Obesity	9.5%	1,588,195	36.3%	575,869	3.8%	21,785	72.6%	15,810	27.4%	5975	***
Comorbidities: Breast cancer	3.8%	627,487	36.7%	230,342	2.3%	5395	79.4%	4281	20.6%	1114	
Comorbidities: Colorectal cancer	1.3%	213,265	32.4%	69,003	2.7%	1853	72.3%	1340	27.7%	513	***
Comorbidities: Prostate cancer	3.8%	638,461	33.7%	215,102	2.3%	4919	74.8%	3679	25.2%	1240	***
Comorbidities: Lung cancer	1.1%	182,387	30.0%	54,775	2.6%	1434	69.5%	997	30.5%	437	***
Comorbidities: Endometrial cancer	0.4%	69,862	35.1%	24,500	2.3%	556	78.1%	434	21.9%	122	
Comorbidities: Anemia	23.2%	3,863,361	32.8%	1,265,982	3.5%	43,845	77.0%	33,762	23.0%	10,083	***
Comorbidities: Leukemia	1.2%	200,409	33.7%	67,496	3.6%	2431	66.8%	1623	33.2%	808	***
Comorbidities: HIV	0.3%	56,390	34.3%	19,354	4.0%	775	73.8%	572	26.2%	203	***
Comorbidities: Transplant	1.5%	243,186	36.3%	88,344	4.0%	3545	64.9%	2302	35.1%	1243	***

Abbreviations: IQR = Interquartile Range; N/A = Not Applicable. † Fully vaccinated: beneficiaries who have been fully vaccinated for COVID-19 before 4 June 2021. Beneficiaries who died before complete vaccination or who were vaccinated with the Pfizer-BioNTech BNT162b2vaccine with less than 20 days between the 2 doses or who were vaccinated with the Moderna mRNA-1273 vaccine and with less than 27 days between the 2 doses were not used in study. ‡ Breakthrough not hospitalized: beneficiaries who had a COVID-19 breakthrough infection but did not require hospitalization for the disease. A breakthrough infection is defined as the first COVID-19 infection after the 1st dose of vaccine for COVID-19 and must happen at least 14 days after complete vaccination. ^¶^ Breakthrough hospitalized: beneficiaries who had a COVID-19 breakthrough infection and required an inpatient admission for management of their disease. Asterisks shown in this section indicate p values for differences in % hospitalization for each characteristic against % hospitalization of breakthrough infections for the total study cohort. ^1^ Calculated with the total Medicare Cohort (65 and over) size as the denominator. ^2^ Calculated with the same row count from the Medicare Cohort (65 and over) population as the denominator. ^3^ Calculated with the same row count from the Fully Vaccinated population as the denominator. ^4^ Calculated with the same row count from the Breakthrough Cases population as the denominator. * *p* < 0.05; ** *p* < 0.01; *** *p* < 0.001 by one-proportion binomial test of difference in % hospitalization for each characteristic against % hospitalization for total study cohort (20.8%). +++ *p* < 0.001 by the Mann–Whitney test of difference between age distribution in the breakthrough cases and in the breakthrough hospitalizations. ^#^ Prior hospitalization means hospitalization before a COVID-19 infection, and therefore applies only to COVID-19 cases. ^##^ Prior COVID means a COVID-19 infection before COVID-19 vaccination and therefore apply only to vaccinated beneficiaries.

**Table 2:** Age Distributions of January & March Vaccinee Groups.

**Fully Vaccinated in January (n = 224,360)**	65 to 74	24%	54,449
	75 to 84	33%	74,585
	85 & Older	42%	95,326
**Fully Vaccinated in March (n = 927,049)**	65 to 74	51%	473,155
	75 to 84	35%	323,098
	85 & Older	14%	130,796

## Data Availability

The study data cannot be made available under the terms of the data use agreement between the U.S. Department of Defense and the Centers for Medicare and Medicaid Services.

## Supplementary Materials

The following are available in the online Supplemental File at https://www.mdpi.com/article/10.3390/biology11121700/s1: Supplemental Methods; Supplemental Results.

## Department of Defense Disclaimer

The views expressed in this article are those of the authors and do not necessarily reflect the official policy or position of the Department of Defense, nor the U.S. Government. Author Peter Walker is a military service member. This work was prepared as part of his official duties. Title 17 USC§105 provides that copyright protection under this title is not available for any work of the U.S. Government. Title 17 USC§101 defines a U.S. Government work as a work prepared by a military service member or employee of the U.S. Government as part of that person’s official duties.
