# Supplementary material for: Enhanced Vaccine Effectiveness during the Delta Phase of the COVID-19 Pandemic in the Medicare Population Supports a Multilayered Prevention Approach
Source: Biology (Basel). 2022 Nov 24;11(12):1700. doi: 10.3390/biology11121700 (PMC9774613; doi:10.3390/biology11121700)
Supplement: Supplementary file 1 [file biology-11-01700-s001.zip › biology-2015327-supplementary.pdf]

Supplementary Materials

Table of Contents:

**S1. Supplemental Methods**	**2–6**
S1.1 *Vaccine Effectiveness Screening Methods*	**2**
S1.2 *Logistic regression model demographic and Insurance Coverage Variables*	**2**
S1.3 *Logistic regression model social Vulnerability Index (SVI) variables associated with zip codes*	**2**
S1.4 *Logistic regression model CMS chronic condition variables*	**2–3**
S1.5 *Logistic regression model ICD-10-CM codes used to identify chronic condition variables*	**3–4**
S1.6 *Logistic regression model Medication variables*	**4–5**
S1.7 *Logistic regression model COVID-19 hospitalization outcome variables*	**5**
S1.8 *Logistic regression model County infection and vaccination rate variables*	**5**
S1.9 *Logistic regression model methods*	**5–6**
**S2. Supplemental Results**	**7–13**
S2.1 Figure S1 *Effect of claim lag on COVID-19 case detection*	**7**
S2.2 Figure S2 *Week at which full vaccination achieved December 2020 – August 2021*	**8**
S2.3 Table S1 *Calculation of imputed COVID-19 breakthrough cases*	**9**
S2.3 Table S2 *Calculation of imputed COVID-19 breakthrough hospitalizations*	**9**
S2.4 Figure S3 *Bar graph of total and breakthrough COVID-19 cases and COVID-19 hospitalizations*	**10**
S2.5 Table S3 *Unvaccinated/Vaccinated COVID-19 infection rate ratios*	**11**
S2.5 Table S4 *Unvaccinated/Vaccinated COVID-19 infection hospitalization rate ratios*	**11**
S2.6 Table S5 *Unvaccinated/Vaccinated COVID-19 infection rate ratios Jan. vs. March vaccinees*	**12**
S2.6 Table S6 *Unvaccinated/Vaccinated COVID-19 hospital. rate ratios Jan. vs. March vaccinees*	**12**
S2.7 Table S7 *COVID-19 Vaccine Effectiveness against infection during the Delta Surge*; Table S8 *COVID-19 Vaccine Effectiveness against hospitalization during the Delta Surge*	**13**

S1. Supplemental Methods

S1.1 Vaccine Effectiveness estimates using screening method and logistic regression fitting

Vaccine Effectiveness for COVID-19 infection and COVID-19 hospitalization was estimated using the screening method as follows:

$$VE = 1 - \frac{PCV}{1 - PCV} \times \frac{1 - PPV}{PPV}$$

where PCV is the proportion of cases (infections or hospitalizations) vaccinated and PPV is the proportion of the population vaccinated.

Vaccine Effectiveness for COVID-19 infection and COVID-19 hospitalization was also estimated using the following linear model:

$$Logit(PCV) = Logit(PPV) + a + \sum_i b_i \times rcs_i(calendar\ week\ \#)$$

where rcs_i are the sub-functions corresponding to the linear tail-restricted cubic spline function used to smoothen the week effect ($i = 4$ by default) and $calendar\ week\ \#$ is the calendar week number the cases are observed on. These models were fitted by logistic regression. The fitting procedure enabled us first to obtain 95% confidence intervals and second to introduce calendar week number as the only covariate of the model. The calendar week number was expanded with linear tail-restricted cubic spline function to get a smoothened evolution of the VE over time (see reference [28] in article). The logistic regression was performed with R statistical software, version 3.6 with the rms package (see references in (see references [29,30] in article).

S1.2 Demographic and Insurance Coverage Variables

Weekly updates of CMS "Master Beneficiary Summary 2020 File" (MBSF_2020) data were processed, extracting variables: ORIG_REASON_FOR_ENTITLEMENT (Disability), ZIP_CD (residential zip code), YOB, SEX_CODE, RACE_CODE, ESRD_INDICATOR and DUAL_STUS_CD 01-12 (Dual Medicare-Medicaid insurance).

S1.3 Social Vulnerability Index (SVI) variables associated with zip codes

SVI values for zip codes were derived from CDC data, which are categorized by census tract. The variables analyzed using binary logistic regression to determine significant predictor variables for hospitalization due to a COVID-19 breakthrough infection included income quartile, education, residential density and other factors using the following SVI variables: the EPL_PCI income > 0.75 (lowest income quartile) variable, the EPL_CROWD > 0.75 (crowded living), EPL_GROUPQ > 0.75 (institutional housing), and EPL_MUNIT > 0.75 (living in multiunit housing).

S1.4 CMS chronic condition variables:

These chronic condition codes found in the MBSF_2020 chronic condition segment file were used in hospitalization and death models:

1. Acute myocardial infarction July-December 2020 (AMI)
2. Chronic kidney disease (CHRONIC_KIDNEY_EVER), COPD (COPD_EVER)
3. Congestive heart failure (CHF_EVER)
4. Diabetes mellitus (DIABETES_EVER)
5. Ischemic heart disease (ISCHEMICHEART_EVER)
6. Stroke/transient ischemic attack (STROKE_TIA_EVER)

7. Breast cancer July-December 2020 (CANCER_BREAST)
8. Colorectal cancer July-December 2020 (CANCER_COLORECTAL)
9. Prostate cancer July-December 2020 (CANCER_PROSTATE)
10. Lung cancer July-December 2020 (CANCER_LUNG)
11. Endometrial cancer July-December 2020 (CANCER_ENDOMETRIAL)
12. Anemia July-December 2020 (ANEMIA)
13. Asthma (ASTHMA_EVER)
14. Hypertension (HYPERT_EVER)

S1.5 ICD-10-CM codes used to identify chronic condition variables (present prior to the COVID-19 diagnosis date). The following chronic condition variables were identified by identifying ICD-10-CM codes for the conditions listed below in these CMS claim files: Part A institutional Inpatient and Outpatient claims, Part B Carrier claims, SNF and Hospice claims from 1 October 2019 received by CMS Chronic Condition Warehouse through 21 August 2021.

S1.5.1 Leukemia (includes acute and chronic myeloid and lymphocytic leukemias as well as less common leukemias) ICD-10-CM codes:
C95, C959, C9590, C9592, C94, C950, C9500, C9502, C92, C929, C9290, C9292, C951, C9510, C9512, C91, C919, C9190, C9192, C93, C939, C9390, C9392, C943, C9430, C9432, C914, C9140, C9142, C901, C9010, C9012, C91A, C91A0, C91A2, C926, C9260, C9262, C92A, C92A0, C92A2, C92Z, C92Z0, C92Z2, C913, C9130, C9132, C916, C9160, C9162, C91Z, C91Z0, C91Z2, C921, C9210, C9212, C93Z, C93Z0, C93Z2, C940, C9400, C9402, C948, C9480, C9482, Z806, C920, C9200, C9202, C910, C9100, C9102, C911, C9110, C9112, C924, C9240, C9242, Z856, C925, C9250, C9252, C915, C9150, C9152, C931, C9310, C9312, C942, C9420, C9422, C933, C9330, C9332, C922, C9220, C9222, C930, C9300, C9302

S1.5.2 Pulmonary fibrosis or pulmonary hypertension (include idiopathic pulmonary fibrosis, interstitial lung disease, pneumoconioses, pulmonary sarcoidosis, pulmonary hypertension) ICD-10-CM codes:
J61, J62, J620, J628, J63, J636, J65, J64, J60, J84, J841, J8417, J848, J849, J8410, J84112, E84, E840, E841, E8419, E848, E849, I270, I272, I2720, I2722, I2723, I2724, D860.

S1.5.3 Chronic liver disease (includes alcoholic cirrhosis, primary biliary cirrhosis, chronic viral hepatitis due to hepatitis B and C, alcoholic fatty liver, primary sclerosing cholangitis, Wilson's disease) ICD-10-CM codes:
K7469, K745, K703, K7030, K7031, P7881, K7460, K74, K743, K744, K746, K717, B18, B180, B181, B182, B188, B189, K70, K709, E8301, K8301, K700, E8801

S1.5.4 HIV/AIDS ICD-10-CM codes:
B20, Z717, O9873, O9872, O98719, O98713, O98712, O98711, O9871, O987, B9735, Z21, Z830

S1.5.5 Transplant (includes following transplants: lung, bone, heart, liver, pancreas, intestine, kidney, bone marrow) ICD-10-CM codes:
Z94, Z949, Z9885, D47Z1, T86810, T86811, T86812, Z942, Z945, Z946, T8621, T8622, T8623, T8641, T8642, T8643, T86890, T86891, T86892, Z941, Z944, Z948, T8611, T8612, T8613, Z940, T86840, T86841, T86842, Z947, Z9483, T86850, T86851, T86852, Z9482, T8631, T8632, T8633, Z9484, T8601, T8602, T8603, T8691, T8692, T8693, Z9481, Z7682, Z943, T86, T8681, T862, T864, T868, T8689, T861, Y830, T8685, T865, T863,

T86818, T869, T860, T8619, T8629, T8649, T86898, T86848, T86819, T86858, T8620, T8639, T8640, T86899, C802, T8609, T8610, T8699, T86849, T86859, T8630, Z4824, T8600, T8690, Z482, Z4821, Z4823, Z4822, I2575, I25750, I25751, I25758, I25759, I25811, Z48280, Z4829, Z48290, Z4828, I2576, I25760, I25761, I25768, I25769, I25812, I257

S1.5.6 Obesity (BMI 30 kg/m^2 - 40kg/m^2) ICD-10-CM codes:
Z6830, Z6831, Z6832, Z6833, Z6834, Z6835, Z6836, Z6837, Z6838, Z6839, E6609, E661, E668, E669

S1.5.7 Morbid obesity (BMI over 40kg/m^2) ICD-10-CM codes:
Z6841, Z6842, Z6843, Z6844, Z6845, E6601, E662

S1.6 Medication variables:
Inpatient, Outpatient, Carrier, Part D, Hospice and SNF Data Files were analyzed by the Humetrix SaaS system to derive the medication variable types 1 - 12 listed below. RxNorm TTY = IN ingredient codes for all pharmaceutical classes are available on request.

1. Chemotherapy: signifies that a beneficiary at any time after 2020 either had an ICD-10-CM code for chemotherapy in part A Institutional or Part B Carrier claims,
    a. had one of the, HCPCS or CPT-4 codes listed or the ICD-10-CM codes in any claim listed below indicating administration of parenteral chemotherapy in a Part B Carrier claim:

       CPT-4 chemotherapy codes:
       96401, 96402, 96405, 96406, 96409, 96411, 96413, 96415, 96416, 96417, 96420, 96422, 96423, 96425, 96440, 96446, 96450, 96542, 96549, G0498

       HCPCS chemotherapy codes:
       J8501, J8510, J8515, J8520, J8521, J8530, J8560, J8562, J8565, J8600, J8610, J8650, J8700, J8705, J8999, J9000, J9010, J9015, J9017, J9019, J9020, J9022, J9023, J9025, J9027, J9032, J9033, J9034, J9035, J9036, J9039, J9040, J9041, J9042, J9043, J9044, J9045, J9047, J9050, J9055, J9057, J9060, J9065, J9070, J9098, J9100, J9119, J9120, J9130, J9145, J9150, J9151, J9153, J9160, J9171, J9173, J9176, J9178, J9179, J9181, J9185, J9190, J9199, J9200, J9201, J9203, J9204, J9205, J9206, J9207, J9208, J9210, J9211, J9212, J9213, J9214, J9215, J9216, J9228, J9229, J9230, J9245, J9250, J9260, J9261, J9262, J9263, J9264, J9265, J9266, J9267, J9268, J9269, J9270, J9271, J9280, J9285, J9293, J9295, J9299, J9300, J9301, J9302, J9303, J9305, J9306, J9307, J9308, J9309, J9310, J9311, J9312, J9313, J9315, J9320, J9325, J9328, J9330, J9340, J9351, J9352, J9354, J9355, J9356, J9357, J9360, J9370, J9371, J9390, J9395, J9400, J9600, J9999

       ICD-10-CM chemotherapy codes:
       D61810, Z5112, Z511, D6481, Z5111, Z511, D701, T80810, T80810D, T80810S, T80810A, D701, D702

    b. had a pharmacy (PDE) claim with an NDC code which mapped to an RxNorm ingredient code for an active pharmaceutical ingredient belonging to multiple classes of chemotherapeutic agents based on the National Cancer Institute list of drugs used in the treatment of cancer (https://www.cancer.gov/about-cancer/treatment/drugs#F). A code of

1 (=True) was only assigned to these variables if a prescription was filled with a sufficient quantity to overlap the date of the first claim with a COVID-19 ICD-10-CM code.

For Variables 2-11, medications were identified by mapping the NDC drug product codes to RxNorm ingredient codes belonging to the indicated pharmaceutical classes of drugs. A code of 1 (=True) was only assigned to these variables if a prescription was filled with a sufficient quantity to overlap the date of the first claim with a COVID-19 ICD-10-CM code.

2. Anticoagulant drugs (VKORC1 and factor X inhibitors)
3. Antiplatelet drugs (cyclooxygenase inhibitors, ADP receptor inhibitors, adenosine reuptake inhibitors, phosphodiesterase inhibitors)
4. Beta-2 agonists
5. Corticosteroids (excludes topical and ophthalmic preparations)
6. Opioid drugs
7. Histamine type-2 receptor blockers
8. Angiotensin converting enzyme inhibitors (ACE inhibitors)
9. Angiotensin II receptor blockers
10. Non-steroidal anti-inflammatory (NSAID) drugs
11. Immunosuppressive drugs of diverse pharmaceutical classes including anti-interleukins, anti-TNFa drugs, JAK kinase inhibitors, anti-interferons, Sphingosine 1-p receptor modulators, calcineurin inhibitors, mycophenolate and sirolimus and methotrexate.
12. Azithromycin and Chloroquine drugs (includes both Chloroquine and Hydroxychloroquine). A PDE claim date no more 10 days beyond the first claim with a COVID-19 ICD-10-CM code was required to identify a beneficiary as taking one of these two drugs and assign a code of 1 (=True).

S1.7 COVID-19 outcome variables:

COVID-19 hospitalizations were identified either by Part B Carrier claims with place of service code = 21 or CPT codes indicating inpatient care with a date of service no more than 14 days after or 10 days before the COVID-19 diagnosis date, or by finding Part A Inpatient claims with an admission diagnosis of COVID-19 and where the data of admission no more than 14 days after or 10 days before the COVID-19 diagnosis date.

- CPT-4 codes indicating inpatient services

99217, 99218, 99219, 99220, 99221, 99222, 99223, 99224, 99225, 99226, 99231, 99232, 99233, 99234, 99235, 99236, 99238, 99239

S1.8 County infection and vaccination rate variables:

Two variables were defined at the individual residential county level, one indicating if the county vaccination rate 14 days before the breakthrough infection was in the top quartile of U.S. counties and the other indicating if the county COVID-19 infection rate 14 days before the breakthrough infection was in the top quartile of U.S. counties.

S1.9 Logistic regression model methods

We used logistic regression to identify significant predictors of COVID-19 breakthrough hospitalization using version 3.6 R statistical software with rms, glmnet and pROC" packages (see references [29-32] in article) and R Studio (Boston, MA, USA). The following binary outcomes were used: beneficiaries who only

received outpatient care; and those who were hospitalized for COVID-19. This predictive model was developed and evaluated on a sample from our Medicare cohort composed of all COVID-19 breakthrough cases with a breakthrough infection date from 6 February 2021, through 10 July 2021. We estimated that this sample is composed of 90% COVID-19 infections due to SARS-CoV-2 strains preceding the appearance of the Delta B.1.617.2 variant mainly including the D614G lineages of the Wuhan strain and the Alpha B.1.1.7 strain of SARS-CoV-2 (see reference [33] in article). Eighty percent of the 16,729 COVID-19 cases hospitalized for COVID-19 in the sample were randomly allocated to the training set used to develop our model, and the remaining 20% went to the validation set used to measure our model's performance. Randomly chosen controls, who received outpatient care only for COVID-19 were included in the training and validation sets such that the case control ratio was 50:50 in the training set and 20:80 in the validation set (the ratio of COVID-19 breakthrough hospitalizations to COVID-19 breakthrough cases we observed in our Medicare cohort). We used a stepwise backward variable selection procedure based on the Akaike Information Criterion (AIC) to remove non-significant variables. Computation of the 95% confidence intervals for the coefficient estimates were generated by bootstrapping (2000 repetitions) on the training set.

The independent variables included in our COVID-19 breakthrough infection hospitalization risk model were: beneficiary age, sex, ethnicity, dual Medicare and Medicaid insurance coverage and residential zip code, prior hospitalization(s) >1 since October 1 2019, COVID-19 infection(s) prior to the first dose of vaccine, the individual's chronic conditions (described in section 1.4 and 1.5), medications grouped by pharmaceutical class as described in section 1.6, residence in a county in the top quartile of U.S. county infection rate and vaccination rate (described in section 1.8) and the following CDC Social Vulnerability Index (SVI) variables: residential zip code income quartile (CDC SVI_EPL_PCI >0.75), residential zip code multiunit housing (SVI_EPL_MUNIT >0.75), residential zip code institutional housing (SVI_EPL_GROUPQ >0.75), predicted from CDC SVI census track based data (see section 1.3).

We also included a variable for the number of days between the second dose of COVID-19 vaccine and the date of diagnosis of the COVID-19 breakthrough infection. This variable was included in the model as a continuous variable with a linear effect.

Variables which did not survive backward variable selection using the Akaike Information Criterion (AIC) procedure to remove non-significant variables included: hypertension, anemia, acute MI in second half of 2021, ischemic heart disease, HIV, diabetes, cerebrovascular disease, Asian and unknown ethnicities, residence in a zip code in the lowest quartile of income (SVI_EPL_PCI variable), crowded housing (SVI_EPL_CROWD variable), institutional housing (SVI_EPL_GROUPQ variable) and highest quartile county vaccination rate; a history of breast, prostate, colorectal, endometrial or lung cancer; as well as prescriptions for Angiotensin 2 receptor blocker drugs, chloroquine, hydroxychloroquine, corticosteroid drugs and H2 blocker drugs overlapping the COVID-19 diagnosis date.

Lung cancer, Asian race and unknown race had P values > 0.10.

S2. Supplemental Results

S2.1 Effect of claim lag on COVID-19 case detection: Estimate of Magnitude of missing COVID-19 cases counts variable intervals between week of observation and Extraction of Claims from CMS Chronic Condition Data Warehouse

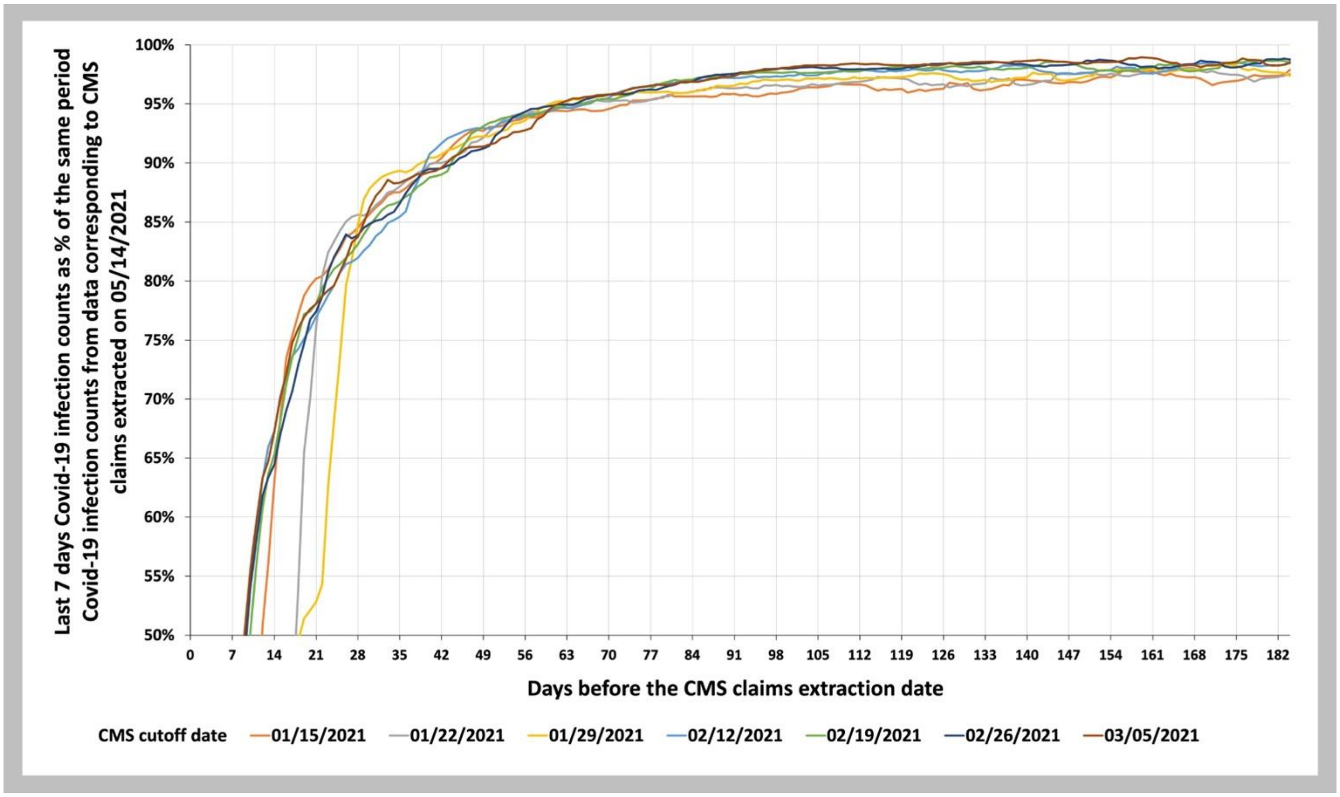

Figure S1 Estimate of Completeness of 7-days COVID-19 Case Counts depending on the number of days between the COVID-19 infection dates and the date of claims extraction from the CMS Chronic Condition Data Warehouse. Each of the 7 curves represents 7-day COVID-19 cases counts calculated from a given extraction of claims from the CMS Chronic Condition Data Warehouse occurring between 01/15/2021, and 03/05/2021. The dates of these extractions are indicated in the key below the x-axis. The 7-days COVID-19 cases counts are expressed as a percentage of COVID-19 cases counts for the same periods calculated from an extraction of claims occurring on 05/14/2021. They are displayed based on the number of days between the observation week of COVID-19 infections and the date of claims extraction.

S2.2 Distribution of dates full vaccination achieved in the 65 and over cohort

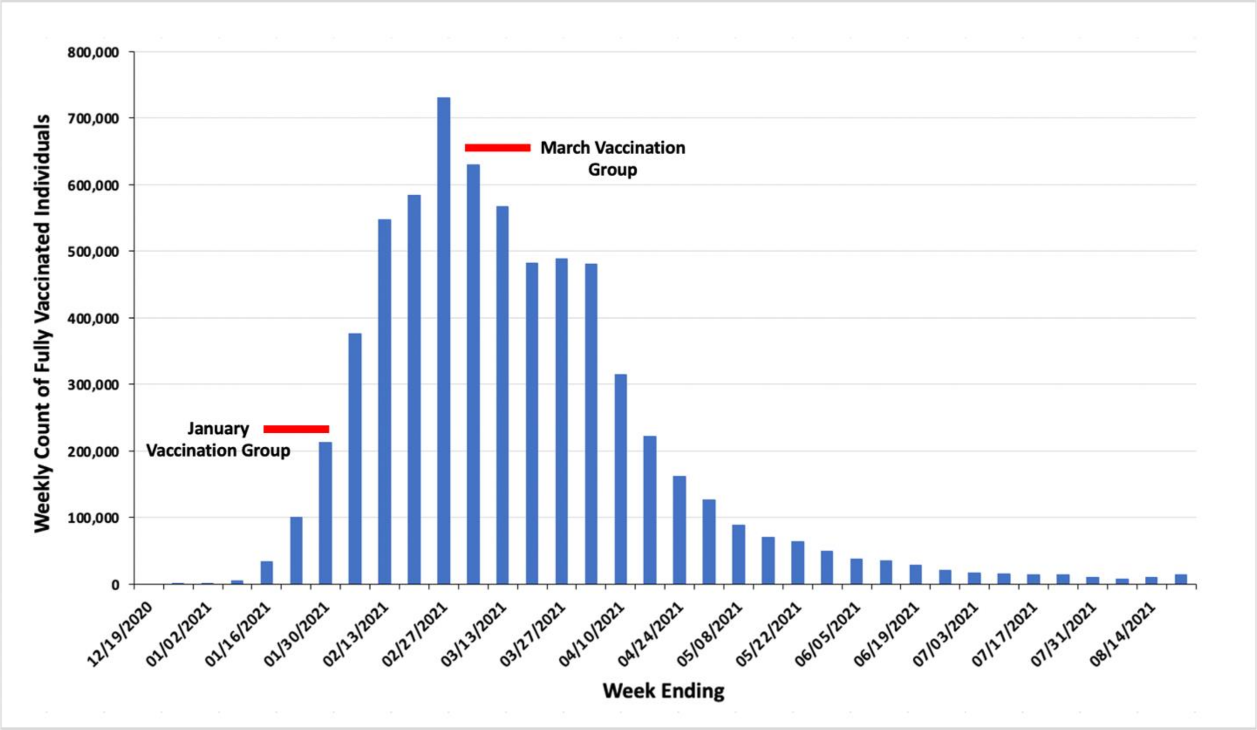

Figure S2 Weeks at Which 65 and Over Years Old Beneficiaries Became Fully Vaccinated. The median day of vaccination is 03/08/2021 (IQR is 02/18/2021 to 03/30/2021); the median week of vaccination is the week ending 03/13/2021 (IQR week ending 02/20/2021 – week ending 04/03/2021). Also shown are the weeks full vaccination was achieved for the January and March vaccinee groups (described in Table 2 in the Results section) used to compare COVID-19 breakthrough infection and hospitalization rates in figures 3, 4 and 5 in the Results section).

S2.3 Calculation of imputed COVID-19 breakthrough cases and breakthrough hospitalizations

Week			65+ Vaccination Rates 14 days before Week Start		Cases		
Week Start	Week End	Calendar Week #	CDC	Medicare Cohort	Total COVID-19 Cases	Unadjusted Breakthrough Cases	Imputed Breakthrough Cases
					181,569	57,655 (32%)	117,411 (65%)
07/04/21	07/10/21	27	77.0%	38.5%	8,672	2,989	5,978
07/11/21	07/17/21	28	77.5%	38.6%	12,479	4,184	8,428
07/18/21	07/24/21	29	78.5%	38.7%	17,994	5,795	11,751
07/25/21	07/31/21	30	78.8%	38.8%	26,915	8,711	17,714
08/01/21	08/07/21	31	79.3%	38.9%	33,327	10,294	20,989
08/08/21	08/14/21	32	79.6%	39.0%	39,495	12,395	25,326
08/15/21	08/21/21	33	79.9%	39.0%	42,687	13,287	27,224

Table S1 Calculation of Imputed COVID-19 Breakthrough Cases. Imputed Breakthrough Cases (column 8) are calculated from Unadjusted Breakthrough Cases (column 7) and by multiplying by the CDC and Medicare Cohort 65+ Vaccination Rate ratios (columns 4 & 5). See section 2.4 “Imputation of COVID-19 breakthrough case and hospitalization counts” in the Materials and Methods section, and below.

Week			65+ Vaccination Rates 14 days before Week Start		Hospitalizations		
Week Start	Week End	Calendar Week #	CDC	Medicare cohort	Total COVID-19 Hosp.	Unadjusted Breakthrough Hosp.	Imputed Breakthrough Hosp.
					41,802	11,399 (29%)	23,164 (55%)
07/04/21	07/10/21	27	77.0%	38.5%	2,935	884	1,768
07/11/21	07/17/21	28	77.5%	38.6%	3,990	1,162	2,342
07/18/21	07/24/21	29	78.5%	38.7%	5,728	1,624	3,293
07/25/21	07/31/21	30	78.8%	38.8%	7,893	2,131	4,333
08/01/21	08/07/21	31	79.3%	38.9%	9,952	2,637	5,377
08/08/21	08/14/21	32	79.6%	39.0%	11,304	2,961	6,050

Table S2 Calculation of Imputed COVID-19 Breakthrough Hospitalizations. Imputed Breakthrough Hospitalizations (column 8) are calculated from Unadjusted Breakthrough Hospitalizations (column 7) and by multiplying by the CDC and Medicare Cohort 65+ Vaccination Rate ratios (columns 4 & 5). See section 2.4 “Imputation of COVID-19 breakthrough case and hospitalization counts” in the Materials and Methods section, and below.

For imputing the number of COVID-19 breakthrough infections and associated hospitalizations, we assumed that the breakthrough infection and hospitalization rates among the vaccinated beneficiaries with missing vaccination data were the same as the breakthrough infection and hospitalization rates among the identified vaccinated beneficiaries in our cohort. For this adjustment, our Medicare cohort vaccination rates were calculated with all the fully vaccinated in our cohort including those vaccinated with Janssen COVID-19 vaccine and the beneficiaries vaccinated with mRNA vaccines regardless of the time elapsed between the first and second dose. These imputed breakthrough case and hospitalization counts were used to calculate infection and hospitalization rates displayed in Figure 1 and to compute VE estimates displayed in Figure 2.

Note: Raw data showed unexpected peaks in numbers of total and breakthrough COVID-19 cases and hospitalizations. These peaks occurred on the Tuesday of each week likely due to post weekend adjustments and were also found in the last day(s) of almost every month during the observation period, in this case believed to correspond to healthcare provider end of month adjustments in the administrative claim submission process. We avoided the weekly spike by summing our study on a weekly basis, and then also manually corrected the monthly peaks first by replacing the unexpected high daily numbers with average numbers based on the surrounding days, and second, by allocating the difference in number of cases and hospitalizations to all the days of the given month weighted by the actual daily numbers of COVID-19 cases and hospitalizations (monthly numbers of COVID-19 cases and hospitalizations remain unchanged).

S2.4 Bar graph of COVID-19 cases and COVID-19 hospitalizations in total cohort and in fully vaccinated beneficiaries

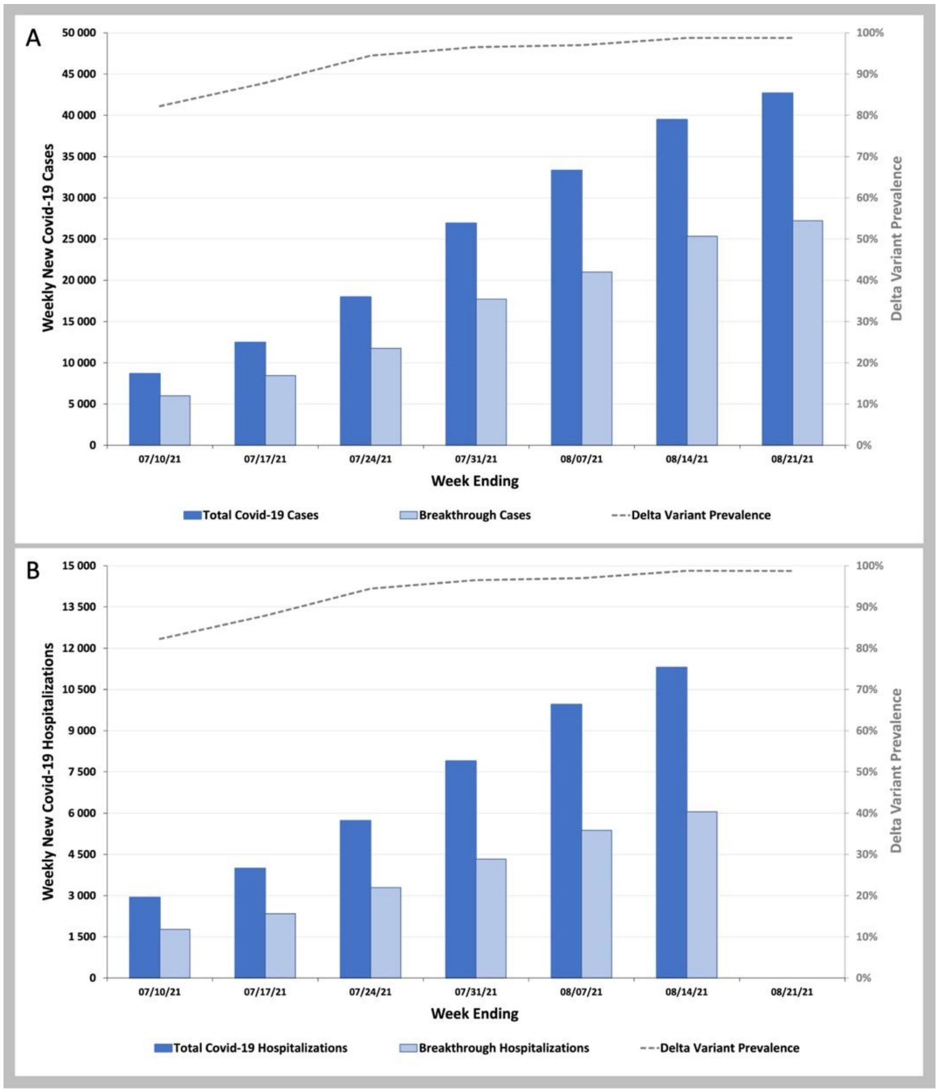

Figure S3 COVID-19 Cases and Hospitalizations in Total Cohort and in Fully Vaccinated Beneficiaries during the Delta Variant Phase of the Pandemic

A - Weekly COVID-19 Cases in Total Cohort (dark blue bars) vs Imputed COVID-19 breakthrough Cases in Fully Vaccinated Beneficiaries (light blue bars).

B - Weekly COVID-19 Hospitalizations in Total Cohort (dark blue bars) vs Imputed COVID-19 Hospitalizations in Fully Vaccinated Beneficiaries (light blue bars).

Weekly SARS-CoV-2 Delta Variant Prevalence reported by the CDC (grey dashed line).

S2.5 COVID-19 infection and COVID-19 hospitalization rate ratios between vaccinated and unvaccinated beneficiaries

Week			Infection Rates per 100K/week		Infection Rate Ratios
**Week Start**	**Week End**	**Calendar Week #**	**Not vaccinated (NV) (Imputed)**	**Vaccinated (V)**	**NV/V**
07/04/21	07/10/21	27	70.2	46.5	1.5
07/11/21	07/17/21	28	107.9	65.2	1.7
07/18/21	07/24/21	29	174.1	89.7	1.9
07/25/21	07/31/21	30	260.2	134.8	1.9
08/01/21	08/07/21	31	357.3	158.7	2.3
08/08/21	08/14/21	32	416.4	190.8	2.2
08/15/21	08/21/21	33	461.2	204.3	2.3

Table S3 COVID-19 Infection Rates in Unvaccinated and in Vaccinated Beneficiaries. Infection Rates in Unvaccinated Beneficiaries (column 4) were imputed. These rates were calculated by subtracting the Imputed Breakthrough Case counts from the Total COVID-19 Case counts. In column 6, the Imputed Infection Rates in Unvaccinated Beneficiaries were compared to the Infection Rates in the Vaccinated Beneficiaries.

Week			Hospitalization Rates per 100K/week		Hospitalization Rate Ratios
**Week Start**	**Week End**	**Calendar Week #**	**Not Vaccinated (NV)**	**Vaccinated (V)**	**NV/V**
07/04/21	07/10/21	27	30.4	13.8	2.2
07/11/21	07/17/21	28	43.9	18.1	2.4
07/18/21	07/24/21	29	67.9	25.2	2.7
07/25/21	07/31/21	30	100.7	33.0	3.1
08/01/21	08/07/21	31	132.5	40.7	3.3
08/08/21	08/14/21	32	154.4	45.6	3.4

Table S4 COVID-19 Hospitalization Rates in Unvaccinated and in Vaccinated Beneficiaries Hospitalization Rates in Unvaccinated Beneficiaries (column 4) were imputed. These rates were calculated by subtracting the Imputed Breakthrough Hospitalization case counts from the Total COVID-19 Hospitalization case counts. In column 6, the Imputed Hospitalization Rates in Unvaccinated Beneficiaries were compared to the Hospitalization Rates in the Vaccinated Beneficiaries.

S2.6 COVID-19 infection and COVID-19 hospitalization rate ratios between beneficiaries vaccinated in January 2012 versus March 2021 and unvaccinated beneficiaries

**Week**	**Infection Rates per 100K**			**Infection Rate Ratios**		
**Calendar Week #**	**Not vaccinated (NV) (Imputed)**	**Vaccinated in January (VJ)**	**Vaccinated in March (VM)**	**VJ/VM**	**NV/VJ**	**NV/VM**
27	70.2	95.9	40.7	2.4	0.7	1.7
28	107.9	173.3	58.2	3.0	0.6	1.9
29	174.1	241.6	86.4	2.8	0.7	2.0
30	260.2	330.8	130.3	2.5	0.8	2.0
31	357.3	313.5	171.5	1.8	1.1	2.1
32	416.4	391.8	195.9	2.0	1.1	2.1
33	461.2	452.1	212.3	2.1	1.0	2.2

Table S5 COVID-19 Infection Rates in Unvaccinated Beneficiaries and in Beneficiaries Vaccinated in January 2021 and in March 2021. Infection Rates in Unvaccinated Beneficiaries (column 2) were imputed. These rates were calculated by subtracting the Imputed Breakthrough Case counts from the Total COVID-19 Case counts. In columns 5 to 7, the different Infection Rates were compared two by two.

**Week**	**Hospitalization Rates per 100K**			**Hospitalization Rate Ratios**		
**Calendar Week #**	**Not Vaccinated (NV) (Imputed)**	**Vaccinated in January (VJ)**	**Vaccinated in March (VM)**	**VJ/VM**	**NV/VJ**	**NV/VM**
27	30.4	32.9	10.2	3.2	0.9	3.0
28	43.9	44.1	16.7	2.6	1.0	2.6
29	67.9	47.8	22.5	2.1	1.4	3.0
30	100.7	71.3	31.5	2.3	1.4	3.2
31	132.5	76.7	39.1	2.0	1.7	3.4
32	154.4	84.7	44.8	1.9	1.8	3.4

Table S6 COVID-19 Hospitalization Rates in Unvaccinated Beneficiaries and in Beneficiaries Vaccinated in January 2021 and in March 2021. Hospitalization Rates in Unvaccinated Beneficiaries (column 2) were imputed. These rates were calculated by subtracting the Imputed Breakthrough Hospitalization counts from the Total COVID-19 Hospitalization case counts. In columns 5 to 7, the different Hospitalization Rates were compared two by two.

S2.7 Vaccine Effectiveness during SARS-CoV-2 Delta Surge

Vaccine Effectiveness against Infection (screening method)				
Calendar Week #	Simple formula Estimate	With logistic regression fitting		
		95% CI Lower	Estimate	95% CI Upper
27	33.7%	28.3%	29.8%	31.4%
28	39.6%	38.1%	39.5%	40.9%
29	48.4%	45.5%	46.5%	47.5%
30	48.2%	50.3%	51.2%	52.0%
31	55.6%	53.0%	53.8%	54.6%
32	54.2%	53.9%	54.8%	55.7%
33	55.7%	54.7%	55.6%	56.5%

Table S7 Estimates of Vaccine Effectiveness against Infection obtained using the Screening Method. Estimates in column 2 were calculated using the Screening Method formula given in part 1.1 of the Supplemental Methods. Estimates and 95% Confidence Intervals bounds in columns 3 to 5 were obtained by fitting the linear model for infections given in part 1.1 of the Supplemental Methods by logistic regression.

Vaccine Effectiveness against Hospitalization (screening method)				
Calendar Week #	Simple formula Estimate	With logistic regression fitting		
		95% CI Lower	Estimate	95% CI Upper
27	54.7%	53.7%	55.7%	57.6%
28	58.7%	57.5%	59.0%	60.4%
29	63.0%	61.8%	63.2%	64.4%
30	67.3%	65.8%	67.0%	68.1%
31	69.3%	68.2%	69.4%	70.6%
32	70.5%	69.4%	70.5%	71.5%

Table S8 Estimates of Vaccine Effectiveness against Hospitalization obtained using the Screening Method. Estimates in column 2 were calculated using the Screening Method formula given in part 1.1 of the Supplemental Methods. Estimates and 95% Confidence Intervals bounds in columns 3 to 5 were obtained by fitting the linear model for hospitalizations given in part 1.1 of the Supplemental Methods by logistic regression.
